# AhMADS6‐Mediated Repression of *AhHD‐Zip* Contributes to Growth Habit Regulation in Cultivated Peanut ( *Arachis hypogaea* L.)

**DOI:** 10.1111/pbi.70760

**Published:** 2026-09-22

**Authors:** Yuanjin Fang, Lei Shi, Boyang Zhang, Han Liu, Xiaobo Wang, Pengyu Qu, Huanhuan Zhao, Lulu Xue, Xiaona Li, Feiyan Qi, Ziqi Sun, Zheng Zheng, Xiaodong Dai, Wenzhao Dong, Bingyan Huang, Xinyou Zhang

**Affiliations:** ^1^ Institute of Crop Molecular Breeding, Henan Academy of Agricultural Sciences Zhengzhou China; ^2^ The Shennong Laboratory Zhengzhou China; ^3^ Key Laboratory of Oil Crops in Huang‐Huai‐Hai Plains, Ministry of Agriculture Zhengzhou China; ^4^ Henan Provincial Key Laboratory for Oil Crops Improvement Zhengzhou China; ^5^ College of Agriculture and Biotechnology Zhejiang University Hangzhou China; ^6^ Hainan Institute Zhejiang University Sanya China

Cultivated peanut ( *Arachis hypogaea* L.) is an allotetraploid oilseed crop of global importance. Peanut growth habit has traditionally been classified into four types erect, bunch, spreading and prostrate and is determined largely by the orientation of the first pair of lateral branches. Because pods develop along these lateral branches, growth habit directly affects peg formation, pod distribution and yield potential. Genetic studies have repeatedly identified loci associated with growth habit traits on chromosome Arahy15 (Pan et al. 2022; Yu et al. 2023). Genome‐wide association studies have also identified candidate genes potentially associated with plant architecture on Arahy13 and Arahy15, including genes encoding a brassinosteroid signalling kinase and a MADS‐box protein, respectively (Lu et al. 2024; Zheng et al. 2024). However, these studies either mapped the relevant traits to broad genomic intervals containing multiple genes or lacked functional validation of specific regulators in peanut.

In our previous study, we identified two major quantitative trait loci (QTL) associated with peanut growth habit, *qGHA15* on chromosome Arahy15 and *qGHA06* on chromosome Arahy06 (Fang et al. 2023). Phenotypic variation explained (PVE) of predicted epistatic interaction between these loci was over 60%, suggesting that they contain coordinated regulatory components.

To fine‐map the previously identified *qGHA15* and *qGHA06*, we genotyped 1775 individuals from a segregating F_2_ population derived from recombinant parental lines. Association analysis of markers within *qGHA15* identified a highly significant peak between *Arahy.ATH5WE* and *Arahy.R9TI2M* (Table S1). By contrast, the candidate interval within *qGHA06* contained only one gene, *Arahy.SC7TJM*, which encodes a homeobox‐leucine zipper protein and was designated *AhHD‐Zip*. To identify the most likely candidate gene within *qGHA15*, RNA sequencing was performed using the tips of the first pair of lateral branches collected at five developmental time points from the erect line N02, spreading line N03 and bunch line N04. Three genes, *Arahy.QV02Z8*, *Arahy.509QUQ* and *Arahy.ATH5WE*, showed consistent differential expression and were subsequently examined by quantitative PCR (qPCR) (Table S2). The relative expression of *Arahy.ATH5WE*, designated *AhMADS6*, was significantly higher in the spreading and bunch lines than in the erect line N02 at all five sampling points, and *Arahy.ATH5WE* was functionally validated in priority among the three genes within *qGHA15* (Figure 1a).

**FIGURE 1:**
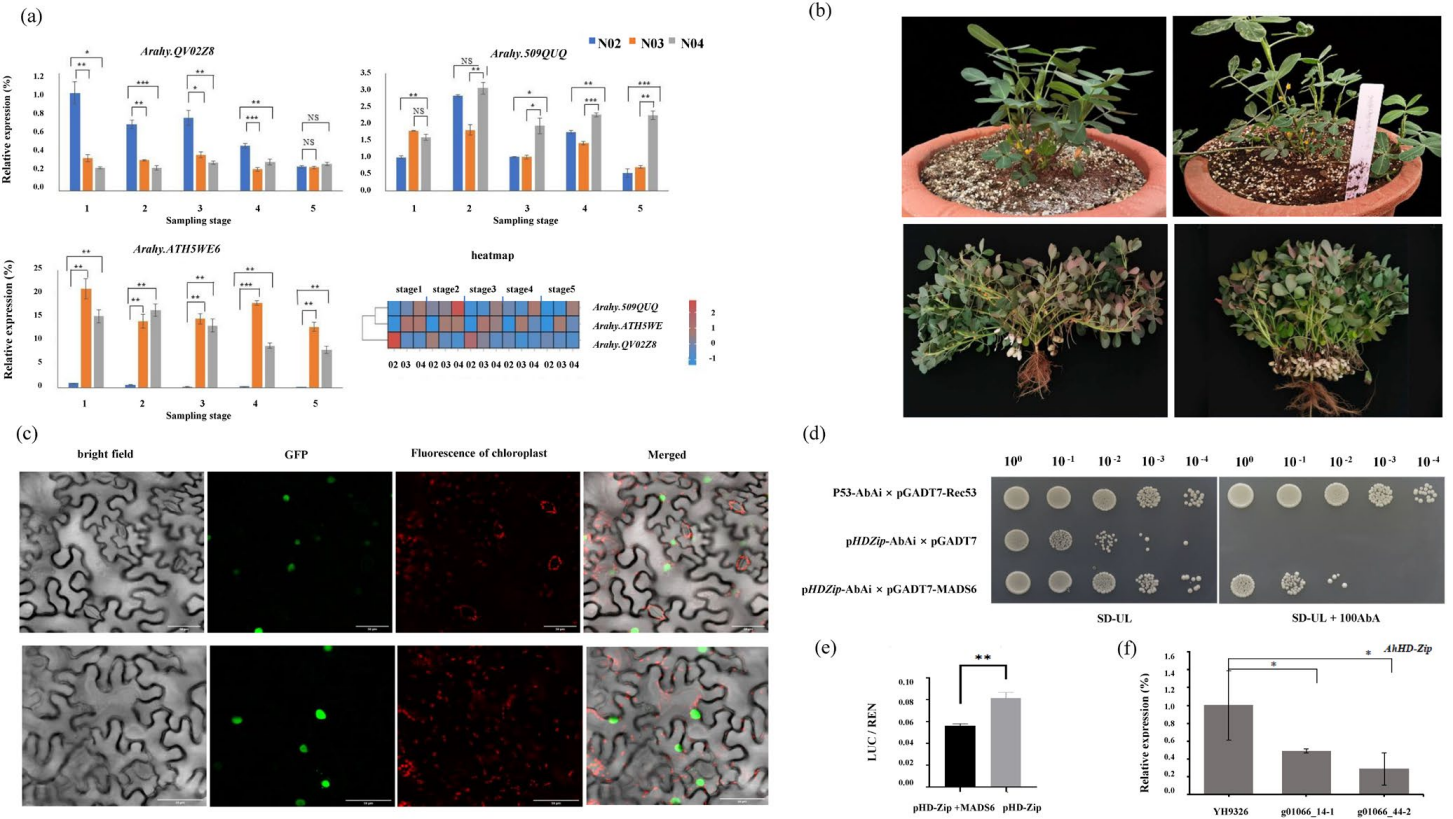
Functional validation of candidate genes associated with growth habit in peanut. (a) Differential expression of candidate genes located within *qGHA15* at five developmental time points in the erect line N02, spreading line N03 and bunch line N04. Data are presented as the mean ± SD of three biological replicates. Statistical significance between genotypes at each time point was evaluated using a two‐tailed Student's *t*‐test, *p < 0.05, **p < 0.01, ***p < 0.001; NS, not significant. (b) CRISPR/Cas9‐mediated disruption of *AhHD‐Zip* alters peanut growth habit. Upper left, the erect wild‐type Yuhua9326; upper right, an *AhHD‐Zip* knockout T_3_ line; lower left, the spreading wild‐type germplasm line PI 290594; and lower right, an *AhMADS6* knockout T_2_ line. (c) Subcellular localization of the AhHD‐Zip protein (upper panel) and AhMADS6 protein (lower panel) fused to GFP in *Nicotiana benthamiana* epidermal cells. Scale bars are shown in the corresponding images. (d) Yeast one‐hybrid assay between pHDZip‐AbAi and pGADT7‐MADS6. (e) dual‐luciferase reporter assay in *Arabidopsis* protoplasts examining the regulatory effect of AhMADS6 on the *AhHD‐Zip* promoter. **p < 0.01, as determined using a two‐tailed Student's *t*‐test. (f) Expression of *AhHD‐Zip* in folded leaves from first pair of lateral branches in two genetic‐edited lines g01066‐14 and g01066‐44 compared to Yuhua9326.

Single‐guide RNAs (sgRNAs) targeting the coding sequences of *AhHD‐Zip* and *AhMADS6* were designed, and CRISPR/Cas9 constructs containing sgRNA‐expression cassettes driven by the *Arabidopsis thaliana AtU6* promoter were generated (Figure S1; Table S3). The construct targeting *AhHD‐Zip* was introduced into the erect cultivar Yuhua9326, whereas the construct targeting *AhMADS6* was introduced into the spreading germplasm line PI 290594. Homozygous mutant lines displaying altered growth habits were subsequently obtained (Figure 1b). Two *AhHD‐Zip* edited lines carried a 3‐bp deletion (‘TCA’) and a 2‐bp deletion (‘TC’), respectively. Growth‐habit‐related traits and pod number per plant differed significantly in the mutant lines (Table S3). To identify vector integration sites and evaluate potential off‐target effects, genomic DNA from mutant lines and wild‐type Yuhua9326 was subjected to whole‐genome sequencing at approximately 20× coverage. Among 2250 predicted potential off‐target sites, two loci in the Tifrunner reference genome (version gnm1.KYV3; Bertioli et al. 2019) exhibited perfect matches to the target sequence. These loci corresponded to the target gene *Arahy.SC7TJM* on Arahy06 (*AhHD‐Zip*) and its homologue *Arahy.QD22BA* on Arahy16 (Table S4). This result was consistent with the gene‐family analysis, which identified 59 *AhHD‐Zip* genes in the peanut genome, and *Arahy.QD22BA* on Arahy16 was the homologue of *Arahy.SC7TJM* (Figure S2). Three additional potential off‐target sites on Arahy06 containing fewer than two mismatches were amplified and validated by Sanger sequencing in two g01066 mutant lines and in wild‐type Yuhua9326 (Figure S3). No novel mutations were detected compared to Yuhua9326. Quantitative PCR analysis of the edited genes in the mutant lines, together with phenotypic evaluation, supported *AhHD‐Zip* as a functional determinant of growth habit in peanut (Figure 1f; Table S3).

The subcellular localization assay showed that both AhMADS6 and AhHD‐Zip proteins localized to the nuclei of *Nicotiana benthamiana* epidermal cells (Figure 1c). Yeast one‐hybrid assays demonstrated that *AhMADS6* could bind to the promoter region of *AhHD‐Zip* using sequence information from the reference genome of the spreading cultivar Tifrunner (Table S5; Figure 1d). To evaluate the transcriptional effect of this interaction in vivo, dual‐luciferase assays were performed in Arabidopsis protoplasts. Co‐expression of *AhMADS6* with the *AhHD‐Zip* promoter‐LUC reporter construct significantly reduced luciferase activity relative to the control treatments (Figure 1e), indicating that AhMADS6 acts as a transcriptional repressor of *AhHD‐Zip*. Quantitative PCR showed that the *AhHD‐Zip* gene was derepressed in the *AhMADS6* mutant background (Figure S4). Collectively, these findings support a regulatory model in which AhMADS6 represses transcription of *AhHD‐Zip* in the spreading cultivar Tifrunner. Nevertheless, the precise AhMADS6‐binding sequence within the promoter remains to be identified. Future studies should also characterize the upstream regulators of *AhMADS6* and the downstream target genes regulated by *AhHD‐Zip* to establish a more comprehensive regulatory network governing peanut growth habit.

In conclusion, this study functionally validates candidate genes located within previously identified interacting QTLs and advances our understanding of the genetic regulation of plant architecture in an allotetraploid legume crop, for which no similar pattern has been reported in other crops. The genes *AhMADS6* and *AhHD‐Zip* therefore represent potential molecular targets for optimizing peanut architecture and improving its suitability for mechanized cultivation.

## Author Contributions

B.H. and X.Z. conceived the study. Y.F., L.S., B.Z., H.L., X.W., P.Q., H.Z., L.X. and X.L. performed the experiments. F.Q., Z.S., Z.Z., X.D. and W.D. provided help with data analysis. Y.F. wrote the manuscript. L.S. and B.H. revised the manuscript. All authors contributed to the article and approved the final manuscript.

## Funding

This work was supported by the National Key Research and Development Project (2022YFD1200400) and the China Agriculture Research System (CARS‐13).

## Conflicts of Interest

The authors declare no conflicts of interest.

## Supporting information


**Figure S1:** CRISPR/Cas9 construct for knocking‐out *AhHD‐Zip* in peanut.

**Figure S2:** Sanger‐sequencing validation of potential off‐target sites in the *AhHD‐Zip*‐edited mutant lines. A06Off1, A06Off2 and A06Off3 represent the selected potential off‐target sites containing fewer than two mismatches on chromosome CM009806.1 position 45624930, 54165646 and 63711918.

**Figure S3:** Cluster analysis of *AhHD‐Zip* gene family. The targeted homologous genes were indicated.

**Figure S4:** *AhHD‐Zip* expression in the lateral branch tip of *AhMAD6*—edited mutant line g01060. PI 290594, g01060 and YH9326 represent spreading type germplasm, erect mutant line and erect variety YU9326 respectively.

**Table S1:** Association analysis of polymorphic markers within *qGHA15* and *qGHA06* in the F_2_ population.
**Table S2:** qPCR primers used to analyse candidate genes associated with peanut growth habit.
**Table S3:** Genotypes and agronomic traits of mutant lines generated through editing of *AhMADS6* and *AhHD‐Zip*.
**Table S4:** Potential off‐target analysis of the mutant line g01066.
**Table S5:** Promoter and coding sequences used in the yeast one‐hybrid assay.

## Data Availability

The RNA‐seq data generated in this study are available in the NCBI Sequence Read Archive under accession number PRJNA1463946.
