# Supplementary material for: AhMADS6‐Mediated Repression of AhHD‐Zip Contributes to Growth Habit Regulation in Cultivated Peanut ( Arachis hypogaea L.)
Source: Plant Biotechnol J. 2026 Sep 22;24(11):5985–7. doi: 10.1111/pbi.70760 (PMC13595818; doi:10.1111/pbi.70760)
Supplement: Supplementary file 1 [file PBI-24-5985-s001.pdf]

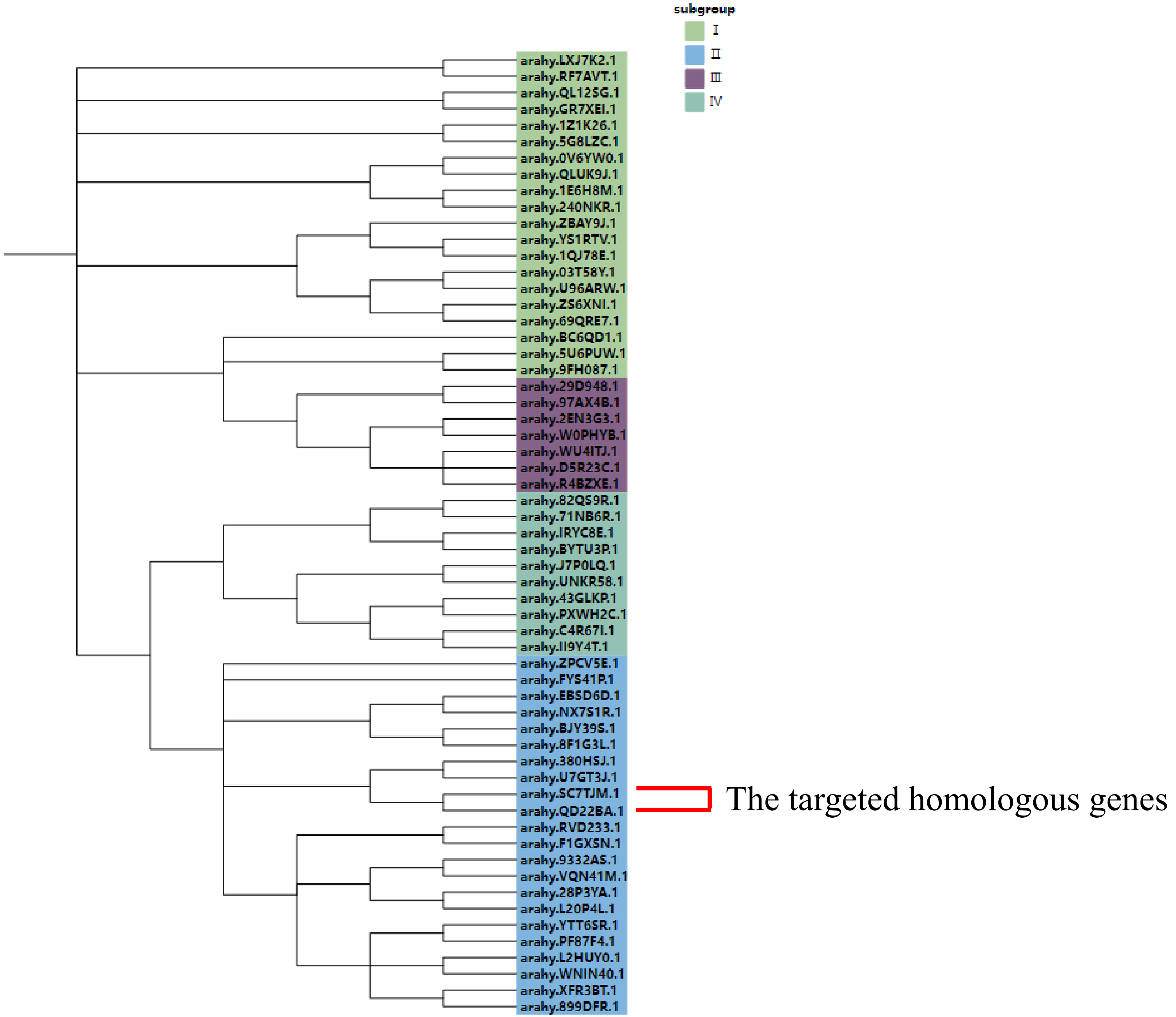
subgroup
I
II
III
IV
arahy.LXJ7K2.1
arahy.RF7AVT.1
arahy.QL12SG.1
arahy.GR7XEI.1
arahy.1Z1K26.1
arahy.5G8LZC.1
arahy.0V6YW0.1
arahy.QLUK9J.1
arahy.1E6H8M.1
arahy.240NKR.1
arahy.ZBAY9J.1
arahy.YS1RTV.1
arahy.1QJ78E.1
arahy.03T58Y.1
arahy.U96ARW.1
arahy.ZS6XNI.1
arahy.69QRE7.1
arahy.BC6QD1.1
arahy.5U6PUW.1
arahy.9FH087.1
arahy.29D948.1
arahy.97AX4B.1
arahy.2EN3G3.1
arahy.W0PHYB.1
arahy.WU4ITJ.1
arahy.D5R23C.1
arahy.R4BZXE.1
arahy.82QS9R.1
arahy.71NB6R.1
arahy.IRYC8E.1
arahy.BYTU3P.1
arahy.J7P0LQ.1
arahy.UNKR58.1
arahy.43GLKP.1
arahy.PXWH2C.1
arahy.C4R67I.1
arahy.II9Y4T.1
arahy.ZPCV5E.1
arahy.FYS41P.1
arahy.EBSD6D.1
arahy.NX7S1R.1
arahy.BJY39S.1
arahy.8F1G3L.1
arahy.380HSJ.1
arahy.U7GT3J.1
arahy.SC7TJM.1
arahy.QD22BA.1
arahy.RVD233.1
arahy.F1GXSN.1
arahy.9332AS.1
arahy.VQN41M.1
arahy.28P3YA.1
arahy.L20P4L.1
arahy.YTT6SR.1
arahy.PF87F4.1
arahy.L2HUY0.1
arahy.WNIN40.1
arahy.XFR3BT.1
arahy.899DFR.1
The targeted homologous genes
