# Supplementary material for: AhMADS6‐Mediated Repression of AhHD‐Zip Contributes to Growth Habit Regulation in Cultivated Peanut ( Arachis hypogaea L.)
Source: Plant Biotechnol J. 2026 Sep 22;24(11):5985–7. doi: 10.1111/pbi.70760 (PMC13595818; doi:10.1111/pbi.70760)
Supplement: Supplementary file 2 [file PBI-24-5985-s002.pdf]

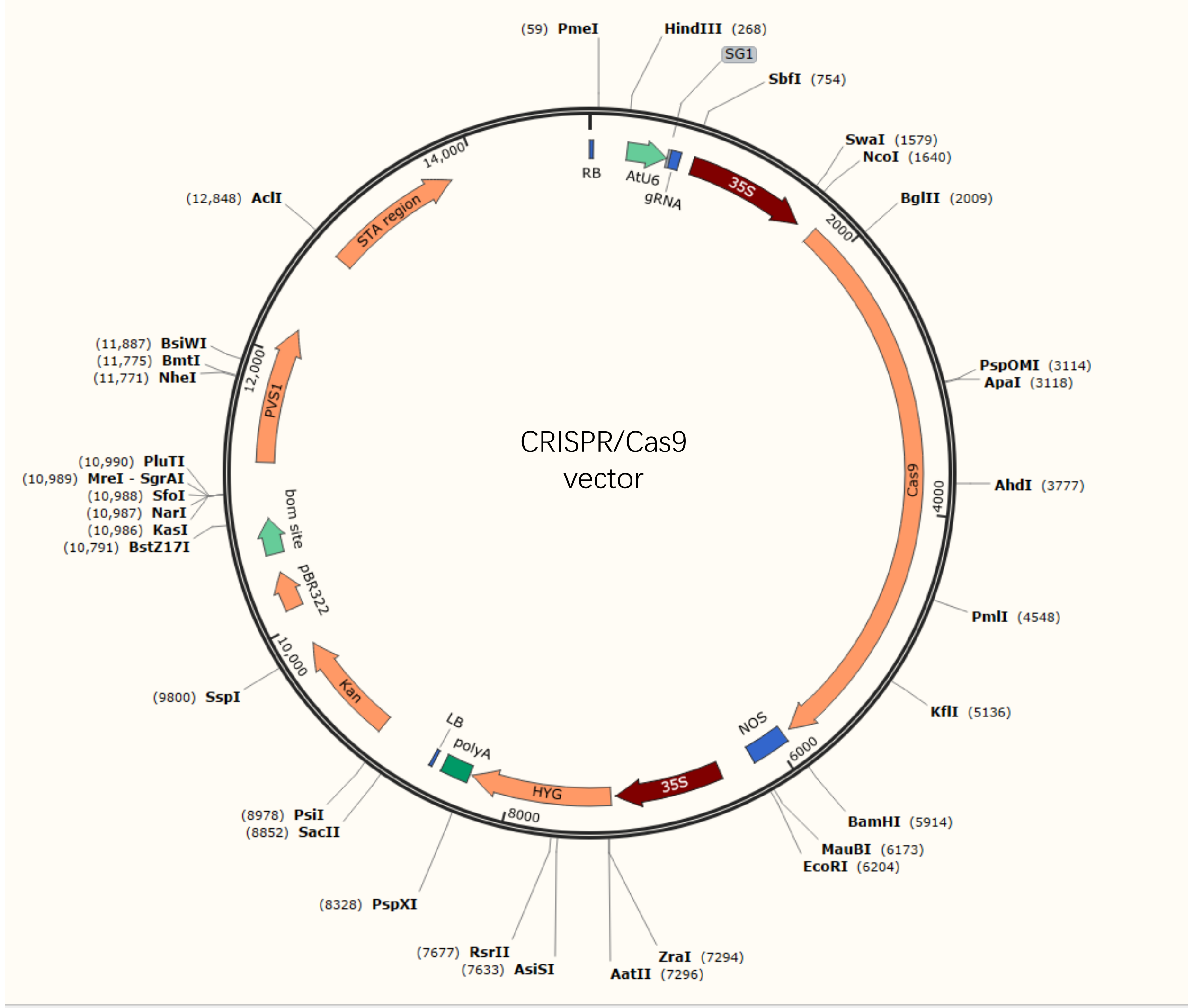
CRISPR/Cas9
vector
(59) PmeI
HindIII (268)
SG1
SbfI (754)
SwaI (1579)
NcoI (1640)
BglII (2009)
PspOMI (3114)
ApaI (3118)
AhdI (3777)
PmlI (4548)
KflI (5136)
BamHI (5914)
MauBI (6173)
EcoRI (6204)
ZraI (7294)
AatII (7296)
(7677) RsrII
(7633) AsiSI
(8328) PspXI
(8978) PsiI
(8852) SacII
(9800) SspI
(10,990) PluTI
(10,989) MreI - SgrAI
(10,988) SfoI
(10,987) NarI
(10,986) KasI
(10,791) BstZ17I
(11,887) BsiWI
(11,775) BmtI
(11,771) NheI
(12,848) AclI
RB
AtU6
gRNA
35S
2000
Cas9
4000
NOS
6000
35S
HYG
8000
polyA
LB
Kan
10,000
pBR322
bom site
PVS1
12,000
STA region
14,000
