# Supplementary material for: AhMADS6‐Mediated Repression of AhHD‐Zip Contributes to Growth Habit Regulation in Cultivated Peanut ( Arachis hypogaea L.)
Source: Plant Biotechnol J. 2026 Sep 22;24(11):5985–7. doi: 10.1111/pbi.70760 (PMC13595818; doi:10.1111/pbi.70760)
Supplement: Supplementary file 4 [file PBI-24-5985-s005.pdf]

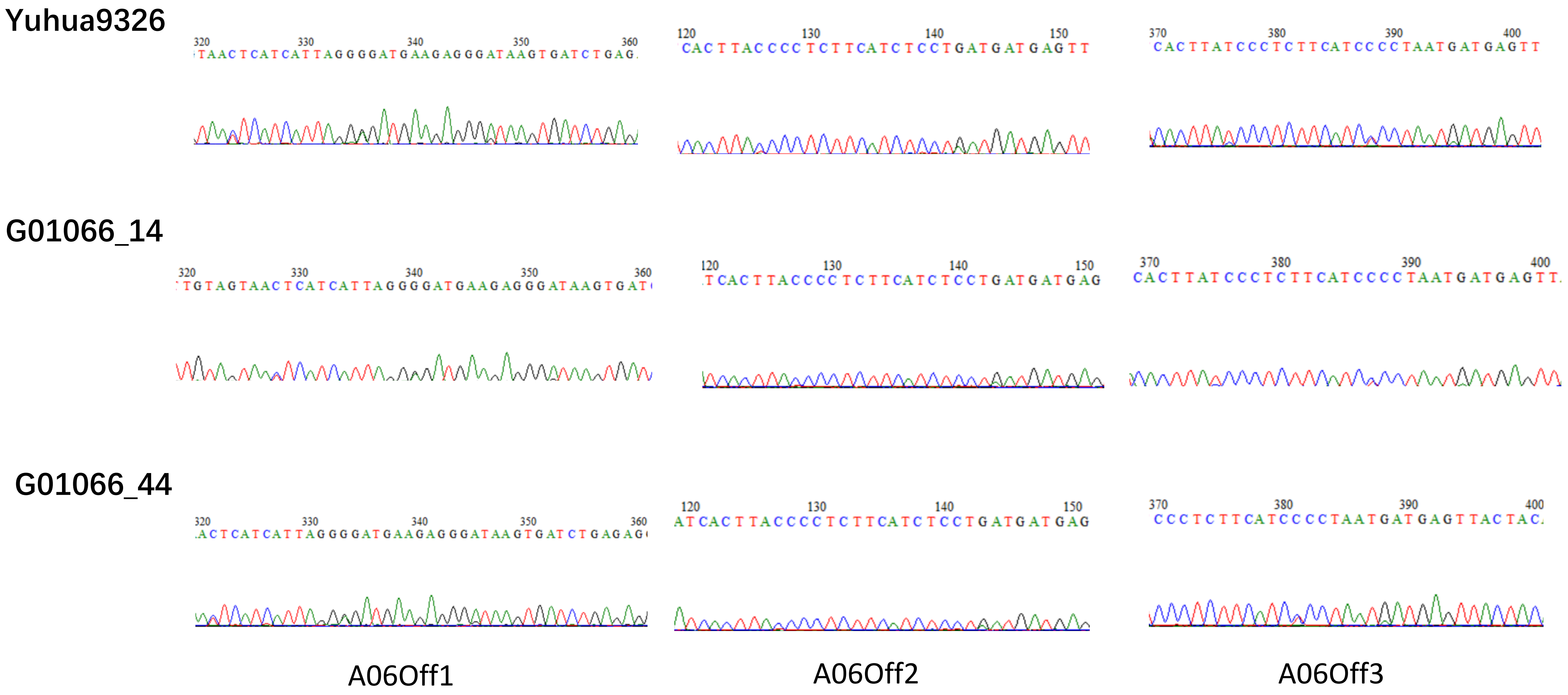

Figure S2 Sanger-sequencing validation of potential off-target sites in the *AhHD-Zip*-edited line g01066
