# Supplementary material for: AhMADS6‐Mediated Repression of AhHD‐Zip Contributes to Growth Habit Regulation in Cultivated Peanut ( Arachis hypogaea L.)
Source: Plant Biotechnol J. 2026 Sep 22:10.1111/pbi.70760. Online ahead of print. doi: 10.1111/pbi.70760 (PMC13595818; doi:10.1111/pbi.70760)
Supplement: Supplementary file 1 [file PBI-9999-0-s002.pdf]

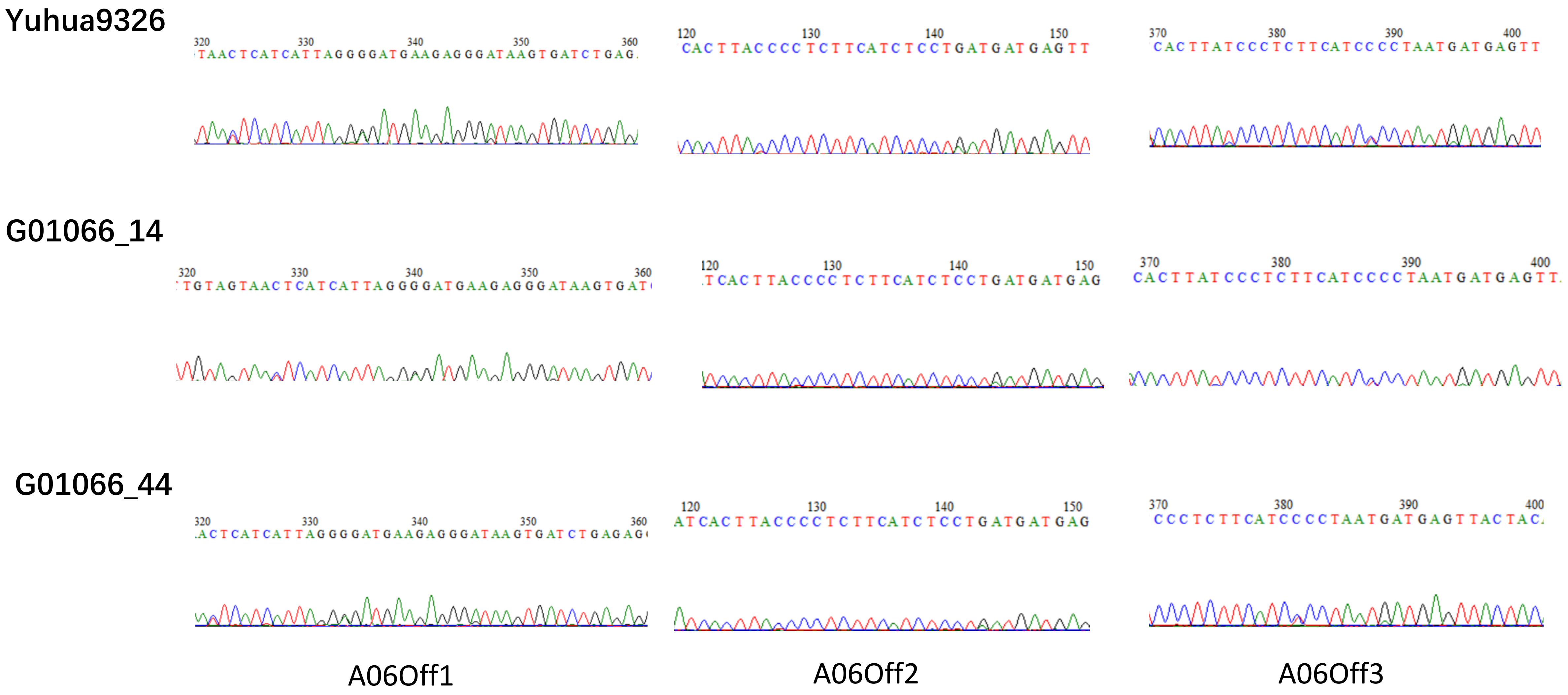

Figure S2 Sanger-sequencing validation of potential off-target sites in the *AhHD-Zip*-edited line g01066
